# Potential of Cold Plasma Technology in Ensuring the Safety of Foods and Agricultural Produce: A Review

**DOI:** 10.3390/foods9101435

**Published:** 2020-10-11

**Authors:** Carolina Varilla, Massimo Marcone, George A. Annor

**Affiliations:** 1 Department of Food Science, University of Guelph, 50 Stone Road East, Guelph, ON N1G2W1, Canada; cvarilla@uoguelph.ca (C.V.); mmarcone@uoguelph.ca (M.M.); 2 Department of Food Science and Nutrition, University of Minnesota, 1334 Eckles Avenue, Saint Paul, MN 55108, USA

**Keywords:** cold plasma, decontamination, food safety, agricultural produce

## Abstract

Cold plasma (CP) is generated when an electrical energy source is applied to a gas, resulting in the production of several reactive species such as ultraviolet photons, charged particles, radicals and other reactive nitrogen, oxygen, and hydrogen species. CP is a novel, non-thermal technology that has shown great potential for food decontamination and has also generated a lot of interest recently for a wide variety of food processing applications. This review discusses the potential use of CP in mainstream food applications to ensure food safety. The review focuses on the design elements of cold plasma technology, mode of action of CP, and types of CP technologies applicable to food applications. The applications of CP by the food industry have been demonstrated for food decontamination, pesticide residue removal, enzyme inactivation, toxin removal, and food packaging modifications. Particularly for food processing, CP is effective against major foodborne pathogenic micro-organisms such as *Listeria monocytogenes* and *Salmonella Typhimurium*, Tulane virus in romaine lettuce, *Escherichia coli* O157:H7, *Campylobacter jejuni*, and *Salmonella* spp. in meat and meat products, and fruits and vegetables. However, some limitations such as lipid oxidation in fish, degradation of the oligosaccharides in the juice have been reported with the use of CP, and for these reasons, further research is needed to mitigate these negative effects. Furthermore, more research is needed to maximize its potential.

## 1. Introduction

In general, plasma is characterized as “the fourth state of matter”. According to Whitehead, J. C. [1], plasma consists of complete or partly ionized gases with a range of charged and neutral reactive species that drive its chemistry. Whitehead, J. C. [1] defined CP as a source of high-energy electrons at ambient temperature and pressure that interact in an open or controlled environment. CP produces reactive species, such as formed molecules, reactive particles, free radicals, UV reactive species, and reactive elements of N_2_, O_2_, and H_2_, either in equilibrium or non-equilibrium state [2]. Thermal plasma on the other hand have reactive species generated in environments or gases that have been heated from a few thousands to more than 10,000 K. In thermal plasma, the reactive electrons and heavier species are normally not in state of equilibrium and the temperatures of electrons produced are even greater than that of the temperatures of heavier species of charged and neutral ions [3]. The heat generated in the production of thermal plasma, however, has a detrimental effect on food.

CP produces high energy at low temperatures by generating excited species in an electric discharge, which creates highly excited species for surface modification and pathogenic microorganism inactivation [4]. The energy from the dissociation of the electrons and the ionization of the background gas is effective in dissociate a wide range of chemical bonds [4].

Typically, CP is generated at 1 atmospheric pressure with electron temperatures generally between 1 and 10 eV [4,5,6]. Whitehead, J C. [1] indicated that several plasma generation parameters, such as plasma reactor design and structure, gas composition, plasma energy, frequency, modulation, pulse form and duration of input energy, are required to produce a well-defined process tailored to specific plasma chemical needs. CP is suitable for food decontamination because it does not include intense system parameters [5,6,7].

**Figure 1:**
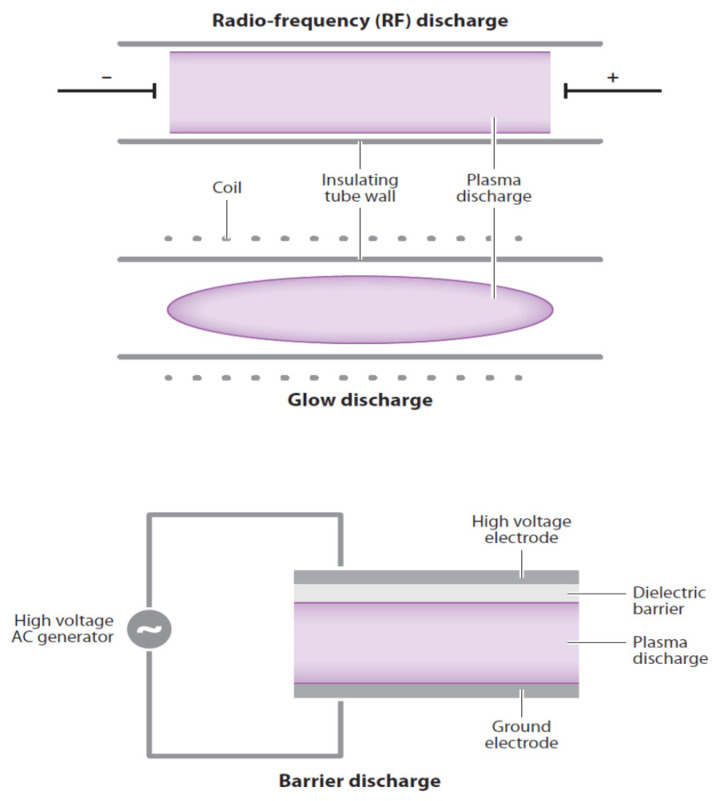
Diagrams of three basic types of discharge: Top—radio frequency discharge, middle—glow discharge, bottom—barrier discharge. Purple colored zones are cold plasma discharge. [8].

There are three basic types of CP discharge systems [8] (Figure 1). First is the non-thermal glow discharge plasma, which is produced by applying a voltage between two electrodes in a glass tube containing a low-pressure gas. The second type involves a radio frequency discharge using pulsed electricity to produce CP in the center of an electric coil. The third type of plasma generation uses an insulating (dielectric) material between electrodes to disperse current flow to generate plasma. The discharge barrier is highly suitable for inactivating microorganisms on fresh produce in packages where reactive oxygen (ROS) and nitrogen (RNS) species can be generated directly within sealed packages [9].

The low heat capacity of atmospheric CP, the diversity of plasma applications, and above all, the cost-effectiveness of its production makes it a very adaptable technology for the food industry. CP kills food pathogens of concern with little to no thermal disruption of food commodities [8]. CP inactivates pathogens by three modes of action [10]. The first mode is the interactions between chemical compounds of reactive molecules, radicals, and positive/negative charge particles and microbes [10]. The next mode is the destruction of the membranes of microorganisms and structural cell functions by UV radiation. The last mode is the breaking of target deoxyribonucleic acid (DNA) by the ultraviolet (UV) radiation emitted by recombining plasma species [10]. The efficacy of inactivating microbes with CP is dependent on these modes of actions and can be optimized for specific food commodities.

It is important to note that the effectiveness of plasma-reactive species for food applications depends on the appropriateness of the products to be treated and the configuration of the CP system. Some of the key factors to consider to effectively decontaminate foods with CP are water activity (commodity and system environment), protein and fat contents, the physical structure of the commodity, spore-forming properties and load of microorganisms. Controlling water activity in sprouted seeds is very critical in removing primary sources of foodborne bacterial pathogen *L. monocytogenes, B. cereus, Salmonella*, and *E. coli* [11]. Significantly different protein and fat content of skinless vs. skin on poultry pieces is an example of how foods with different amounts of proteins and fats contents can result in two distinctly different efficacies with respect to surface disinfection with CP [12]. CP treatment may have also an effect on the color, taste, texture, and aroma of fresh commodities such as blueberries [13,14,15,16,17,18].

## 2. Design Elements of Cold Plasma Technology

CP works under ambient pressure or in a partial vacuum. Many specific gases, such as oxygen, nitrogen, or a combination of noble gases, such as helium, argon, or neon can be used. The high energy produced in CP from direct current, low frequency, or radio frequency voltage of 40 KHz, 13.56 MHz is usually applied over a pair or multiple electrodes [19]. CP systems for use in food sterilization can be divided into three general device configurations [20]. Each system set-up generates different antimicrobial efficacy depending on the interaction between the target surface and the generated plasma. These three different systems setups are based on the positioning of the plasma generating source and the target surface. They are (a) remote treatment, (b) direct treatment, and (c) Close proximity with one of the electrodes.

**Figure 2:**
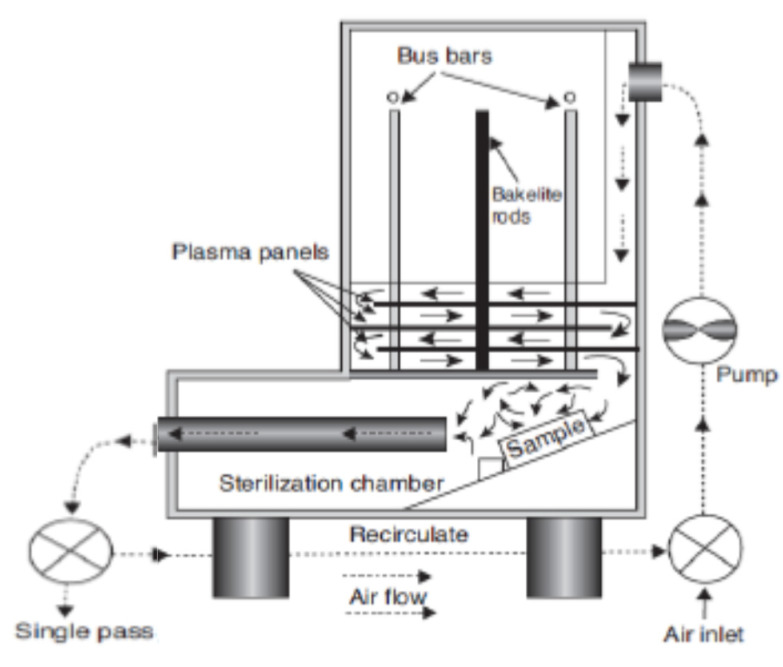
Remote exposure plasma generator [22].

**Figure 3:**
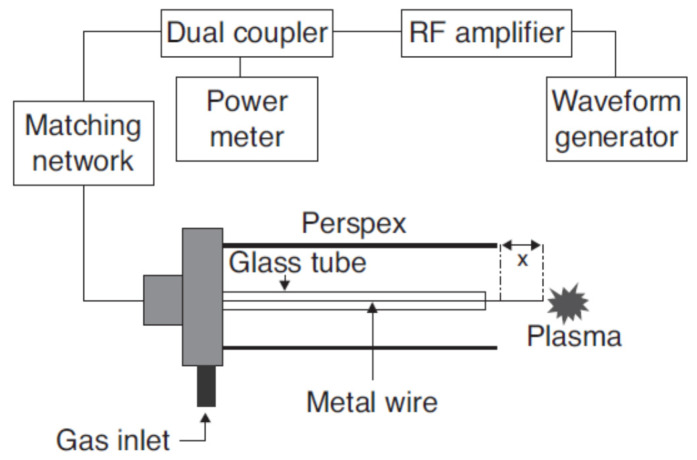
Plasma needle [25].

**Figure 4:**
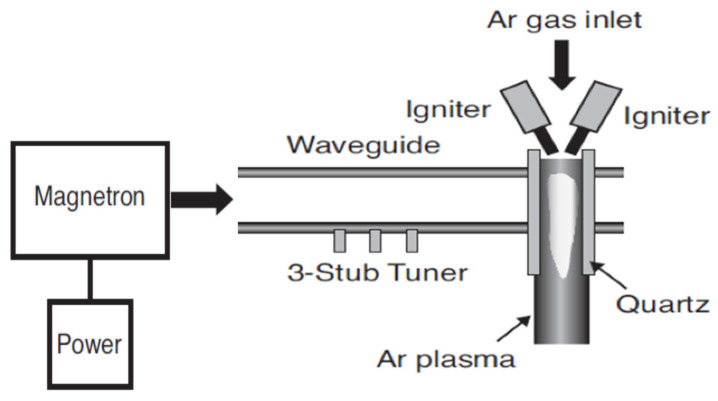
Microwave plasma tube [26].

In the remote treatment, the target product is not directly placed in the chamber where the plasma is being produced (Figure 2). This type of design has some advantages in terms of its design simplicity and flexibility of the size and physical shape of the target product. The gas being ionized may be air or nitrogen, or a mixture of noble gases with electricity, microwave, or laser [21]. According to Gadri, Roth, Montie, Kelly-Wintenberg, Tsai, Helfritch, Feldman, Sherman, Karakaya, and Chen [22], the drawback of this device setup is that the reactive species generated in the generation chamber [23] can interact with other plasma species, including either with charged particles or photon species, resulting in lower secondary energy and thus lower potential for microbial inactivation. In the direct treatment CP systems (Figure 3 and Figure 4), the generated plasma is directly in contact with the target product. The plasma needle (Figure 3) produces 2–3 mm of CP at the tip of a thin, straight steel tube, which is a 1 cm diameter coaxial hollow cylinder [24,25]. The feed gas was mainly He (about 0.3 L/minute) and radio frequency (0.2–0.5 kV, 13.56 MHz) with a total power consumption of approximately 20 mW to 3W depending on the parameters of treatment [24,25]. The microwave plasma tube (Figure 4) [26] is fed with argon (at 100 L/minute) in a microwave system (1 kW, 2.45 GHz). The UV intensity produced at the point of treatment varied from 65 to 94 W/cm^2^.

The mechanism of plasma inactivation of microbes in this setup gives a much higher efficacy because of the direct proximity to the target (i.e., a higher concentration of reactive species compared to the remote treatment [20]). This set-up avoids or minimizes the recombination of generated reactive species with other ions and atoms.

Because this system gives a much higher degree of UV radiation exposure due the close proximity of the target product, there is the possibility of heat generation by conduction in products with high moisture contents or water activity [20]. This will have detrimental effects on the surface of some meat or plant products (i.e., burnt marks or protein coagulation, as well as subtler scent, texture, appearance, and deterioration of other vitamins and nutrients). It is important to note that this system can be flexible and can also accommodate different sizes and shapes of products to be treated based on the specificity of the plasma emitter [8].

**Figure 5:**
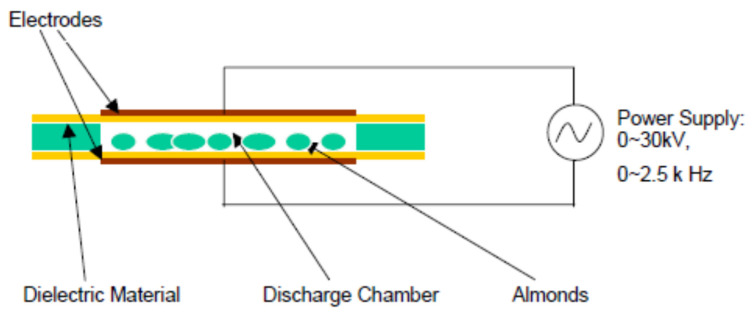
Dielectric barrier discharge [28].

**Figure 6:**
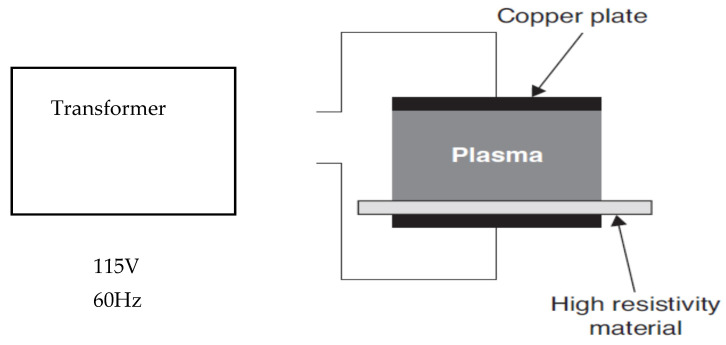
Resistive barrier discharge [29].

The third plasma system is where the products to be treated are in very close contact with one of the electrodes [22] (Figure 5 and Figure 6). The target products in this setup are exposed to the greatest combination of reactive plasma species and also to the highest possible free molecular species concentration, reactive charged particles, negative charged electrons, and UV radiation [27]. In order to avoid point discharges and localized heating issues, it is very important to carefully choose plasma parameters suitable for the size and shape of the products to be treated [8]. According to Deng, Ruan, Kyoon Mok, Huang, and Chen [28], suggested that the target product to be sterilized must fit between the electrodes. Although there are physical constraints in the space between the electrodes in this type of electrode system setup, versatility arises in the ability to change the inlet gas composition and the design parameters of the electrodes [22]. This setup is best suited for smaller products (seeds, berries, nuts, etc.) and flatter objects (chicken breast) [22].

## 3. Mode of Action of CP and Its Use in Food Decontamination

The principal active components identified for the antimicrobial activity of CP systems are reactive oxygen species (ROS). Xu, Shen, Zhang, Ma J., Ma R., Zhao, Sun, Qian, Zhang, Ding, Cheng, Chu, and Xia [30], reported that the generation of reactive oxygen species within the cells of microorganisms results in their inactivation, particularly *Staphylococcus aureus*. Ali, Kim, Lee J. Y., Lee S., Uhm, Cho, Park, and Choi [31] established a negative relationship between ROS formation and spoilage bacteria inactivation. Lee, Park, Jin, Kim, Han, Kim, Hyun, Chung, and Park [32] also found UV radiation as the predominant sterilizer when microorganisms are exposed to high UV radiation levels. CP also produces hydrogen peroxide and nitrogen species which have antimicrobial properties [33]. Patil, Moiseev, Misra, Cullen, Mosnier, Keener, and Bourke [9], and Dorai, and Kushner [34], identified that free radicals viz, hydroxide, peroxide, and oxides of nitrogen were generated by electrical discharges in humidified air. While small amounts of ozone were detected, its content diminished as humidity levels increased. Hydroxides plays a major role in sterilization in aqueous media and is created by CP from the separation of water molecules. Equations (1)–(3) [35] demonstrate the production of hydroxide radical by plasma.

O + H2O → 2OH(1)

H2O + O3 → O2 + 2OH(2)

H2O + e → OH + H + e(3)

From the predominant species of reactive oxygen species (ROS) and UV radiation to free radical species, hydroxides, peroxides, and oxides of nitrogen and ozone, the decontamination properties of CP have been observed to kill (‘inactivate’) bacteria by destroying the surface of the bacteria through a lethal combination of these CP reactive species.

CP has been shown to be effective in the inactivation of pathogens on the outer layer of fresh products. Table 1 provides a brief overview of the efficacy of CP treatment for the inactivation of various foodborne microbes. Studies by Albertos, Martín-Diana, Cullen, Tiwari, Ojha, Bourke, Álvarez, and Rico [36] on the effect of dielectric barrier discharge (DBD) plasma on fresh mackerel (*Scomber scombrus*) fillet showed that CP is a possible alternative for reducing spoilage bacteria, such as lactic acid bacteria, pseudomonas, and aerobic psychrotrophic bacteria in fish. The authors [36] also reported that CP was effective in reducing the spoilage bacteria in oily fish and that the plasma voltage and time were important parameters for microbial inactivation. However, they also found that CP treatment of fish accelerated lipid oxidation and suggested future studies to investigate the reduction of lipid oxidation by combining the use an antioxidants and CP.

Bußler, Herppich, Neugart, Schreiner, Ehlbeck, Rohn, and Schlüter [13] researched on the impact of cold atmospheric plasma pressure on pea’s physiology and glycoside flavonol (*Pisum sativum ′Salamanca*′). They found that using CP could potentially modified valuable secondary plant metabolites in agricultural plant products. Furthermore, they observed that CP inactivated pathogenic microorganisms on heat-sensitive foods. CP treatment, however, decreased the photosynthetic capacity of seedlings but increased the concentration of flavonoid glycosides in peas, which they attributed to high levels of UV radiation and exposure time. The authors [13] suggested the possible use of CP technology during the post-harvest processing of agricultural products for the selective alteration of important secondary plant metabolites. 

Grzegorzewski, Ehlbeck, Schlüter, Kroh, and Rohn [37], studied the impact of treating lamb lettuce (*Valerianella locusta*) with a CP and its influence on phenolic profile. Exposing lamb lettuce to CP resulted in a negative impact on phenolic acids and flavonoids. Treatment with CP resulted in a substantial reduction of phenolic acid in lamb lettuce. The reduction in phenolic acid was, however, slower compared to the degradation of flavonoids by CP. They [37] suggested that the interaction of different reactive plasma species caused the phenolic acid and flavonoids to degrade but not due to changes in photo- or thermal desorption at the lettuce surface. While there was a reduced level of phenolic acid, a strong increase in diosmetin was detected in the plasma treatment of *Valerianella locusta*.

Min, Roh, Niemira, Sites, Boyd, and Lacombe [38] examined the effect of dielectric barrier discharge (DBD) atmospheric CP treatment on the deactivation of Tulane virus, *Escherichia coli* O157: H7, *Salmonella*, and *Listeria monocytogenes*. Romaine lettuce inoculated with *Listeria monocytogenes*, *Salmonella*, *E. coli* O157: H7, and Tulane virus was packaged in modified atmospheric packaging (MAP), petri dish, and pouched leaves, and flushed with O_2_ at 5% or 10% nitrogen. Packaged lettuce samples were then treated with CP atmospheric dielectric discharge at 34.8 kV for 5 min and then analyzed at 4 °C either immediately or after storage for 24 h. DBD CP treatment inhibited *E. coli* O157: H7 by approximately 1.1 log CFU, *L. monocytogenes* approximately 1.0 log CFU, and Tulane virus approximately by 1.3 log PFU/g. However, the authors [38] found that MAP was not appropriate for atmospheric CP treatment generated by dielectric barrier discharge due to the interaction of N_2_ and O_2_ that was added packaging.

Oh, Song, and Min [39] examined the effects of microwave CP treatment on *Salmonella typhimurium* inhibition and the characteristics of radish sprouts. In the CP microwave generator, nitrogen gas was used at a power of 900 W and pressure of approximately 667 Pa for 1–20 min. Optimized treatment at 10 min. led to a reduction of 1.8 log CFU/g counts of *Salmonella typhimurium*. Appearance and aroma were however not affected negatively. After treatment with CP at 4 °C to 10 °C, radish sprouts stored in enclosed plastic barrier packages had no *Salmonella typhimurium* growth, and also did not affect the water quality, color, antioxidant activity, or ascorbic acid of the sprouted radish. 

The authors concluded that CP had the potential to be used as a tool to inactivate *Salmonella typhimurium* on radish sprouts without altering sensory attributes. According to them, storing the radish sprouts after treatment with CP in closed plastic moisture barrier packages at cold temperatures was beneficial and should be suitable for other vegetable sprouts.

Ziuzina, Misra, Cullen, Keener, Mosnier, Vilaró, Gaston, and Bourke [18] researched on the antimicrobial efficacy of an open-air dielectric discharge barrier plasma reactor (SAFEBAG test device, Dublin Institute of Technology, Dublin, Ireland) on *Escherichia coli* and *Listeria innocua* and also indigenous microflora of cherry tomato after packaging. Cherry tomato quality was also assessed during extended shelf storage. After a continuous cycle of atmospheric CP treatment, reduction of *E. coli* and *Listeria innocua* on average of (3–5) log CFU/g was observed. Using the static mode, maximum reductions of 3.5 log CFU/g of indigenous microflora (mesophiles, yeast, and mold) were achieved. No substantial variations were reported in mean in color (L, a *, and b *), firmness, pH, and total soluble solids. They concluded that a pilot scale in-package CP system may perhaps stimulate discharges into food packages during static and continuous CP treatment methods. The authors also concluded that factors such as bacteria type (Gram-positive and Gram-negative), cell concentration, and mode of treatment influenced decontamination efficacy and should be of importance to achieve an optimized treatment in any industrial settings of CP technology.

The inhibitory effects of atmospheric CP in addition to the use of a surfactant and lactic acid on *monocytogenes*, and *E. coli* producing verotoxins in red chicory was studied by Trevisani, Berardinelli, Cevoli, Cecchini, Ragni, and Pasquali [40]. Red chicory was pre-washed with a solution containing a surfactant, lactic acid, or NaCl, then rinsed with deionized water before CP treatment. CP treatment parameters were fixed at 19.15 V and 3.15 A for 15 min. The authors [40] found that pre-washing red chicory with a surfactant before CP treatment reduced VTEC counts by 2.89 log CFU/g and pre-washing with lactic acid + surfactant gave a much higher VTEC counts reduction of 4.78 log CFU/g. Reduction in counts of *L. Monocytogenes* greater than 4 log CFU/g were observed following red chicory pre-washing with lactic acid and a surfactant. No detrimental effects on color, flavor, or quality were observed. Odor and overall acceptability decreased marginally during storage. They concluded that the synergistic effect of the combination of surfactant, lactic acid, and CP treatment improved the overall bactericidal efficacy. Pre-washing with a surfactant and lactic acid reduced the number of free-flowing bacteria.

With the aim of decontaminating radicchio leaves, Pasquali, Stratakos, Koidis, Berardinelli, Cevoli, Ragni, Mancusi, Manfreda, and Trevisani [16] investigated the efficiency of dielectric barrier CP discharge on *E. coli* O157: H7 and *L. monocytogenes*. Leaves were qualitatively assessed for microbial reduction, antioxidant activity, and sensory characteristics. Microbial reduction of *E. coli* O158:H7 (approximately 1.4 log10 CFU/g) and reduction of *Listeria monocytogenes* (approximately 2.0 log10 CFU/g) after 15 min and 30 min, respectively, were observed. According to the above-mentioned authors, the longer treatment for *L. monocytogenes* was similar to other reported studies using also atmospheric CP dielectric barrier discharge system. Furthermore, Gram-positive bacteria (*L. monocytogenes*) were less susceptible to atmospheric CP compared to Gram-negative bacteria (*E. coli* O158:H7). No major reduction in antioxidant activity was observed in the radicchio leaves after CP treatment. The authors [16] suggested enhanced equipment configurations of the atmospheric CP system were needed to reduce the negative effect on the nutritional quality (such as a significant decrease in Chroma C* color and sensory characteristics after storage).

Min, Roh, Niemira, Boyd, Sites, Uknalis, and Fan [41] studied the inactivation effectiveness of CP on *Escherichia coli* O157:H7. They also investigated dielectric discharge barrier CP effects on surface morphology, brightness, and dioxide generation of bulk Romaine lettuce. Dried pre-washed cut leaves were sealed in a commercial clamshell container assembled in three rows with either 1, 3, 5, or 7 layers of stacked leaves. The authors [41] reported that atmospheric dielectric barrier discharges CP inhibited *Escherichia coli* O157: H7 in a number of stack layers. From their analysis *Escherichia coli* O157: H7 was inhibited in the 1, 3, and 5-layer stacked leaved, but not in the 7-layer stacks. The authors concluded that container headspace volume was an important factor when considering sealed containers for treatment. Having a headspace volume in a sealed container allowed CP reactive species to be diffused equally within the sample. No major changes were found in the particle size, brightness, oxygen saturation, and mass reduction of the leaves.

The antimicrobial efficacy of an open-air dielectric plasma discharge reactor on *Escherichia coli* O157: H7, *Bacillus cereus*, *Bacillus subtillis* on brown rice was reported by Lee, Kim, Woo, Jo, Kim J, Kim S, Park, Oh, and Kim [15]. They reported that CP treatments could improve the microbial decontamination of brown rice. Consistent with other study findings [16], Gram-positive *Bacillus cereus*, and *Bacillus subtillis* took longer to inactivate compared to Gram-negative *Escherichia coli* O157:H7. Plasma treatments effectively improved water absorption and activity of α-amylase but decreased brown rice pH and hardness. The authors [15] concluded that the use of CP technology was effective in maintaining microbial safety and improved the textural quality of brown rice.

Lacombe, Niemira, Gurtler, Fan, Sites, Boyd, and Chen [14] studied the inhibition of aerobic bacteria on blueberries with CP and its impact on quality attributes. The research was conducted to determine the efficacy of CP in decreasing total aerobic count, yeast/molds, total anthocyanin, and blueberries firmness. Treatments with CP significantly lowered the number of microflorae on fresh blueberries stored at 4 °C for 7 days. Significant reductions in compression firmness after 60 s and anthocyanin after 90 s of CP treatment were observed. In this research, CP treatment was unsuccessful in reducing yeast and mold counts. In addition, extended treatment impacted the surface color (L* and a*, b*values). The authors [14] concluded in their study that atmospheric CP can inactivate microorganisms on blueberries on an experimental scale. They also proposed that this technology be applied at the pilot and commercial scales incorporating traditional forced air-cooling processes for optimized decontamination in blueberries and other small fruits.

Sarangapani, O′Toole, Cullen, and Bourke [17] assessed the effect of CP on agrochemicals on blueberries. The study also examined any major effects on essential nutritional and physical quality attributes. Boscalid and Imidacloprid were the two specific pesticides used for this study. For this study, a plasma discharge reactor with a high-voltage dielectric barrier was used. Applying 80 kV and 5 min CP treatment, the pesticide reduction potency was roughly 80% for boscalid and 75% for imidacloprid. They [17] predicted that the key species for pesticide degradation were ozone and hydroxyl radicals. The substantial increase in total flavonoids and phenol content of blueberries after 1 min. did not result in any profound effects on physicochemical properties such as color and firmness. However, a longer dose of plasma treatment significantly decreased ascorbic acid. The authors concluded that CP, while retaining some attractive nutraceutical properties, was also a promising technique for agro-chemical reduction and microbial deactivation on fresh produce.

Dirks, Dobrynin, Fridman, Mukhin, Fridman, and Quinlan [42] examined the efficacy of CP dielectric barrier discharge plasma decontamination of microbes on the exterior of skinless deboned chicken breast and chicken thigh with skin. Antibiotic -resistant strains of *Salmonella enterica* and *Campylobacter jejuni* were injected on the skinless deboned chicken breast and skin-on chicken thigh and treated at ambient pressure with CP. The total maximum reduction of *Salmonella enterica* and *Campylobacter jejuni* in chicken thigh and chicken breast were observed for both samples after 3 min of plasma treatment. Levels of 1.3 to 1.8 log CFU on the chicken thigh and around 2.5 log CFU on the chicken breast were observed. They also noticed a decrease in the composition microflora on the chicken breast and thigh after just 30 s of treatment. However, a few limitations using this non-thermal dielectric barrier discharge plasma system on poultry were observed. First was the flat surface requirement, which was difficult to achieve with chicken breast and chicken thighs. The limited size of the probes and the reaction of energetic particles with neutral reactive oxygen resulting in oxidation of lipids was also a disadvantage.

Kim, Yong, Park, Choe, and Jo [43] published a paper on the efficacy of dielectric barrier discharge plasma on pathogen inhibition, sensory attributes, and physicochemistry of raw pork loins. Antibiotic-resistant strains of *E. coli* O157:H7 and *L. monocytogenes* at levels of 101 to 104 CFU/g were injected and also spread on the prepared pork loin. The dielectric barrier discharge plasma gases used were He and He + O_2_ at a duration of 5- and 10-min. *E. coli* growth was reduced to 0.55 log CFU/g following 10 min of plasma treatment using He and He + O_2_. *Listeria monocytogenes* counts was also reduced to 0.59 log CFU/g when the samples were exposed to 10 min. No evident changes in color was observed, but a significant decrease in the pH was observed. In addition, major reductions in sensory quality parameters were observed in plasma-treated samples. It was concluded that the dielectric barrier discharge plasma device had the potential to be used to decontaminate pork loins by inactivating foodborne pathogens, but further research was required to minimize the sensory deterioration.

Kim, Lee, Choi, and Kim, [44] examined the application of radiofrequency (RF) atmospheric pressure plasma to inactivate *Staphylococcus aureus* on beef jerky and its impact on its nutritive and sensory attributes. Argon was applied as inlet gas at a flow rate of 20,000 sccm with a power of 200 W. After about 10 min of plasma treatment of 300 °C, the amount of *Staphylococcus aureus* measured decreased by (3–4) log CFU/g sample. The composition of fatty acid, color, and pure strength of the beef jerky samples was not substantially modified. It was concluded that the radiofrequency atmospheric pressure plasma succeeds in inhibiting *Staphylococcus aureus* with few variations in nutritional and sensory properties beef jerky.

## 4. The Evolving Capability of Cold Plasma (CP) Applications in Spores Deactivation

The evolving capability of cold plasma (CP) applications to inactivate different endospores has already been documented in multiple studies [45,46,47,48,49,50]. A literature review by Boudam, Moisan, Saoudi, Popovici, Gherardi, and Massines [45] on atmospheric pressure cold plasma discharge for bacterial spore inactivation reported that reactive chemical species and UV radiation controlled the spore inhibition process. In addition, to some degree, on the surface of spores, oxygenated species deactivated spores through erosion. When UV radiation was significant, there was enhanced etching action involving UV photons and oxygenated species (known as UV-assisted material etching) [45]. In their paper [45], they concluded that spore inactivation can be successfully accomplished either by dominant UV radiation or by the single interaction of the reactive species.

The lethal effect of atmospheric pressure RF (27.12 MHz)-argon gas-induced plasma jet on *Bacillus atrophaeus* spores and vegetative *Escherichia coli* was examined by Brandenburg, Ehlbeck, Stieber, Woedtke, Zeymer, Schlüter, and Weltmann [46]. Samples of the microorganisms were inoculated on a polyethylene plate (32 × 8 mm^2^) and were in direct contact with the plasma generated. *Bacillus atrophaeus* could still be identified on 2 of the 5 plates after 7 min of CP treatment. For vegetative *Escherichia coli*, there were no microbial growth after 240 s of plasma jet treatment. The authors concluded that maximum reduction factors were achieved through direct cold plasma treatment of bacteria species along with bacterial spores. In addition, in CP treatment, UV radiation and heat application contributed to lower reduction factors.

Hertwig, Reineke, Ehlbeck, Knorr, and Schlüter [47] studied *Bacillus subtilis* spores, *Bacillus atrophaeus* spores, and *Salmonella enterica* decontamination with two types of CP systemson whole black pepper. Direct plasma treatment with a radio frequency (RF) plasma jet and a remote treatment with a microwave generated plasma were used. The authors [47] reported that remote CP treatment inactivated *S. Enterica* and *Bacillus spores*. A much lower inactivation rate was shown by the direct CAPP treatment, likely due to multiple inhibition and the hard surface properties of the pepper corns. *Bacillus subtilis* spores were more tolerance to both CP systems.

A study of Kim, Lee, and Min [48] on *B. cereus* spores on red pepper powder with CP treatment showed no decrease in *B. cereus* spores inoculated in red pepper powder. The author concluded that further studies were needed to analyze the complex parameters of gases, energy, device configurations, and surface characteristics of the sample in order to fully understand the ability of CP to deactivate of spores.

The influence of various process gas compositions on the inactivation effect of a plasma jet generated at atmospheric pressure on bacillus spores was studied by Lassen, Nordby, and Grün [49] and Reineke, Langer, Hertwig, Ehlbeck, and Schlüter [50]. Both studies showed the impact of the composition of process gas and how they influences the sporicidal inactivation of bacterial spores. The above authors [50] found sporicidal effect was produced using argon/H_2_ (RF)-plasma with a 15% argon mixture. When argon with an admixture of oxygen (0.135 percent vol.) and nitrogen (0.2 percent vol.) was used, the authors [49] established a highly sporicidal result. Lassen, Nordby, and Grün [49] also claimed that the inactivation of spores was driven by the action of UV photons with high UV intensity and that the inactivation of spores, in the absence of UV, was dominated by the action of reactive and metastable species.

## 5. Emerging CP Treatment for Removal of Mycotoxin

A journal review on the efficacy of the CP technique for the removal of mycotoxin in food was published by Gavahian and Cullen [51]. Mold species, which produce mycotoxins, are abundant and can grow under a wide range of environmental conditions. The degree of contamination of mycotoxins on food varies according to location, climatic conditions, and other adverse biological influences [52]. Several factors such as geographical region, environmental factors including such extreme temperature, moisture, and humidity levels, chemical substrate content, bacterial competition, and insect disruption, affect the natural fungal growth and consequently, the production of mycotoxins [53]. It is estimated that one-quarter of the world’s main crops are infected with mycotoxins to some degree [53,54]. Mycotoxins are categorized as the most significant chronic dietary risk factor higher than environmental pollutants, agricultural contaminants, chemical additives, or pesticide residues [54,55]. Animals eating mycotoxin-contaminated animal feed will produce meat and milk that contains toxic residues and are metabolized into more toxic compounds [56]. Aflatoxin B1 has been described by the International Cancer Research Agency (IARC) as being carcinogenic to humans. Animal cancer studies have shown that cancer is caused by aflatoxin M1 in the same way as aflatoxin B1. Internationally, regulatory authorities have set a guideline for the maximum permissible amount of mycotoxin in food and animal feed. For many foods, aflatoxins are relatively stable and resistant to breakdown throughout thermal treatment, such as sterilization, ultra-high temperature processing, cooking, and even in refrigerated temperature [57]. Toxins Deoxynivalenol, Zearalenone, and T–2 are produced by species of Fusarium that often infect grains and related products in the field or during storage. Chemically, T–2 toxins and deoxynivalenol fall under the trichothecenes class of mycotoxins. Deoxynivalenol is possibly the main and most widespread grain contaminant [58]. Deoxynivalenol is prevalent in food and feed crops worldwide, including in Canada and the United States. Like other trichothecenes such as T–2 toxin, deoxynivalenol is regarded as food toxin [59]. As mentioned earlier, many mycotoxins are resistant to thermal treatment such as cooking and pasteurization and the potential of using CP for mycotoxin degradation will ensure the safety of grain-based food products. CP studies have shown potential inhibition of fungal growth and degradation of mycotoxins. The study of peanuts, cereals for human and animal consumption, malted barley seeds, maize, hazelnuts, and date palm has shown the potential of CP for fungal inactivation and degradation of several mycotoxins [60,61,62,63,64,65,66]. The emerging use of CP for fungi inactivation and mycotoxin degradation will be very useful in ensuring mycotoxin-free globally.

## 6. Limitation and Negative Impacts of CP

The negative effects of CP treatment on the sensory and nutritional characteristics of treated foods indeed possess a challenge to the development of CP processes and hardware configurations. The presence of reactive oxygen species (ROS) in CP triggers lipid oxidation in meat tissue and fish which negatively affects the acceptability and shelf-life by lipid degradation and oxidative rancidity development. CP also results in the degradation of by extremely polymerized oligosaccharides in juices after extensive CP treatment. Almeida, Cavalcante, Cullen, Frias, Bourke, Fernandes, and Rodrigues [67] suggested that oligosaccharide degradation was mainly by ozonolysis. Ozonolysis triggered glycoside bond cleavage, leading to macromolecule depolymerization and functional group oxidation. Nonetheless, the degradation of the oligosaccharides after CP treatment was sufficient to be classified as prebiotics. Further experiments are needed to be performed to better understand the degradation mechanism of oligosaccharides through CP application and ozone production. A more complete understanding of the mechanisms of lipid oxidation and oligosaccharide degradation is needed if CP is to be used for mainstream food applications. 

## 7. Government Regulations Regarding Food Safety 

Ten federal statutes and 23 sets of laws are implemented by the Canadian Food inspection Agency (CFIA). These laws and regulations govern the protection and quality of food that is sold in Canada and encourage a sustainable resource base for animals and plants. CFIA shares many of its key roles with other departments and agencies of Canadian federal government, regional, territorial, and local authorities, the private sector, and other stakeholders. The CFIA works with its partners to: enforce food safety measures; manage the hazards, incidents, and emergencies of food, animals, and plants; and support the production of food safety and disease control systems to ensure the safety of high-quality Canadian agriculture, livestock, aquaculture, and fishery products. Decisions at the CFIA are focused on high-quality, accurate, and science-based research. Through knowledge, guidance, risk management, the impact of international standards, research and development, and testing informs policy development and program design and implementation [68]. With regard to new emerging technologies, high pressure processing (HPP), microwave heating (MWH), and ultraviolet light (UV) have been the key technologies used and expected for the next 5 years globally. In 10 years, cold plasma (CP) and pulsed electric filings (PEF) are predicted to be more relevant in Europe, while HPP, microwave, and UV remained more relevant in North America [69].

## 8. Summary of the Studies and Future Research

Many of the studies included in this review have shown that CP treatment demonstrated effectiveness in microbial inactivation. While the majority of the CP systems discussed in this review were all lab-scale set-ups, the potential to introduce this application in a food production plant is feasible. However, negative impacts with respect to the sensory attributes and nutritive values of some food commodities have been observed after treatment with CP. This observation was due to some of the current limitations of CP application, especially given that the technology is product specific and results in a very complex series of chemical interactions. During CP treatment of food products, the generated plasma can be too close to, or unevenly distributed around, the product that can affect the physical and functional attributes. CP generates a high electric field, high energy plasma reactive species, and high concentrations of UV radiation, and thus product position and exposure time are critical.

Regardless of the particular techniques used, it has become apparent that with some alterations and improvement of CP, improved results are obtained. In general, the use and chemistry of CP are very complex, and plasma chemistry and dynamics need to be better understood for various technical applications [70]. It is important to be able to identify new plasma sources for process analysis, as well as to monitor and fine-tune the myriad of species, including electrons, ions, radicals, and photons for successful modifications of the attributes of food decontamination and food quality [70].

## 9. Conclusions

CP is an important antimicrobial technology for microbes such as *Staphylococcus aureus*, *E. coli* O157: H7, *Listeria monocytogenes*, *Salmonella Typhimurium* biofilms, and *Bacillus aureus*. Though there are drawbacks of CP treatment in foods, such as speeding up lipid oxidation and having adverse effects on sensory characteristics, CP has been shown to be very efficient alternative method for food decontamination and should be approved as an alternative technology for the decontamination of foodborne pathogenic microorganisms in food commodities.

## Figures and Tables

**Table 1:** Summary of the effects of cold plasma (CP) treatment on food commodities.

Commodity	Plasma Type	Plasma Parameters	Physicochemical Effects	Microbial Effects	References
Peas	Dielectric barrier discharge	6–12 kV/20 MHz/5 min/electrode distance was 20 mm	Decreased the photosynthetic efficiency of seedlings and modified the concentration of flavonoid glycosides	-	[13]
Blueberries	Atmospheric pressure plasma jet	47 kHz/549 W/Air	Significantly reduced firmness after 60 s	Reduced the population of native microflora on fresh blueberries	[14]
			Significantly reduced in firmness. Impacted the surface color.	Was not effective in reducing yeast and mold counts.	
Brown rice	Dielectric barrier discharge	15 kHz/250 W/5–20 min./air/electrode distance was 2 cm.	Decrease in pH and hardness	Reduced bacteria by approximately 2.3 log_10_ CFU/g *Bacillus cereus, Bacillus subtilis*, *E coli O157:H7,* and total aerobic bacteria counts were significantly reduced	[15]
			Increased L* and decreased a* and b* values of brown rice		
Red chicory (radicchio)	Dielectric barrier discharge	15 KV/12.5 kHz/15–30 min/1.5 m/s/air/electrode distance was 0.15 cm	Did not significantly affects the antioxidant activity and external appearance of produce	Reduced *E. coli* O158:H7 (1.35 log_10_ MPN/g)/*L. monocytogenes* (2.2 log_10_ CFU/g) counts	[16]
Blueberries	Dielectric barrier discharge	80 kV and 1 A for 5 min./electrode distance was 35 mm	Significantly decreased of firmness	-	[17]
			Changed color of blueberries but was not statistically significant from untreated blueberries		
			Decreased total polyphenolic content, total flavonoid, ascorbic acid, and anthocyanin at higher voltage and long exposure time.		
			Significantly degraded pesticide residues such as Boscalid and Imidacloprid		
			Removal efficiencies of 75% for Boscalid and 80% for Imidacloprid were observed		
Cherry tomatoes	Dielectric barrier discharge	100 kV/150 sec/air/electrode distance was 4.5 cm	Did not significantly affect color, firmness, pH, and total soluble solids.	Reduced *E. coli* and *Listeria innocua* counts by 3.5 log CFU/g	[18]
				Reduction in mesophiles, yeast and mold by 3.5 log units	
Fresh mackerel	Dielectric barrier discharge	80 kV/5 min/electrode distance was 35 mm	Lipid oxidation occurred	Reduced total aerobic psychrotrophic, *Pseudomonas*, and lactic acid bacteria counts	[36]
			There were no changes in color.		
Lamb lettuce	Atmospheric pressure plasma jet	27.12 MHz/2 min./argon	Had negative impacts on phenolic acids and flavonoids.		[37]
Romaine lettuce	Dielectric barrier discharge	47.6 kV and 1 A for 5 min./electrode distance was 30 mm	Did not result in any signs of burns, wilting, and color.	*E. coli* was reduced by 1.1 log CFU/g	[38]
				Modified atmospheric packaging (MAP) help reduced the inactivation rates of *E. coli*.	
				*Salmonella* was reduced by 0.6 log CFU/g	
				*Listeria monocytogenes* was reduced by 0.8 log CFU/g	
				Tulane virus was reduced by1.3 log PFU/g lettuce with or without evaporation of water.	
Radish Sprouts	Microwave plasma	2.45 GHz/900 W/669 Pa/10 min/N_2_	Did not negatively affect appearance, odor, ascorbic acid, and antioxidant activity	Reduced the number of *Salmonella typhimurium* by 1.8 log CFU/g	[39]
			Radish sprout exhibited wilting after 20 min of plasma treatment.		
Red chicory	Dielectric barrier discharge	19.15 V/3.15 A/15 min/electrode distance was 2.0 cm	No detrimental effects on color, freshness, and texture.	Reduced *L. monocytogenes* by more than 4 log CFU/g	[40]
			Odor and overall acceptability slightly decreased during storage	Reduced VTEC *(E. coli) by* more than *5 log_10_*	
Romaine lettuce	Dielectric barrier discharge	42.6 kV/1.5 A/10 min/air/electrode distance was 5.0 cm	No significant changes in the surface morphology, color, respiration rate, and weight loss were observed	Reduced *E. coli* O157:H7 (0.4 – 0.8 log_10_ CFU/g) in certain layer configurations.	[41]
				Reduction in bulk stacking with 7 layers (1.1 log_10_ CFU/g)	
Skinless chicken breast and chicken thigh with skin	Dielectric barrier discharge	30 kV and 1 A for 3 min.	Decreased nutritional quality, and shelf life due reactive oxygen species that induced lipid oxidation.	Reduced *Salmonella enterica* and *Campylobacter jejuni* counts by 1.3 to 1.8 log CFU/g on skin and approximately 2.5 log CFU/g on breast for both	[42]
				Reduced background microflora	
Raw pork loin	Dielectric barrier discharge	3 kV, 30 kHz/10 min./3 mm between DBD actuator and sample/He or He + O_2_	Reduced pH	Reduced E. *coli* at 0.55 log CFU/g and *L. monocytogenes* at 0.59 log CFU/g	[43]
			Changed meat color		
			Decreased nutritional quality, and shelf life due to reactive oxygen species that induced lipid oxidation.		
			Significantly affected appearance, color, odor, and acceptability.		
Beef jerky	Radio-frequency atmospheric pressure plasma	20,000 sccm /200 W/3 min./argon	Little changes in nutritional qualities such as fatty acid composition and sensory qualities were observed	Reduced *Staphyylococcus aureus* by (3–4) log CFU/g	[44]
